# Supplementary figures and images for: A cocktail of SARS-CoV-2 spike stem helix domain and receptor binding domain human monoclonal antibodies prevent the emerge of viral escape mutants
Source: bioRxiv. 2025 Oct 20:2025.10.16.682699. Preprint. [Version 1] doi: 10.1101/2025.10.16.682699 (PMC12633237; doi:10.1101/2025.10.16.682699)

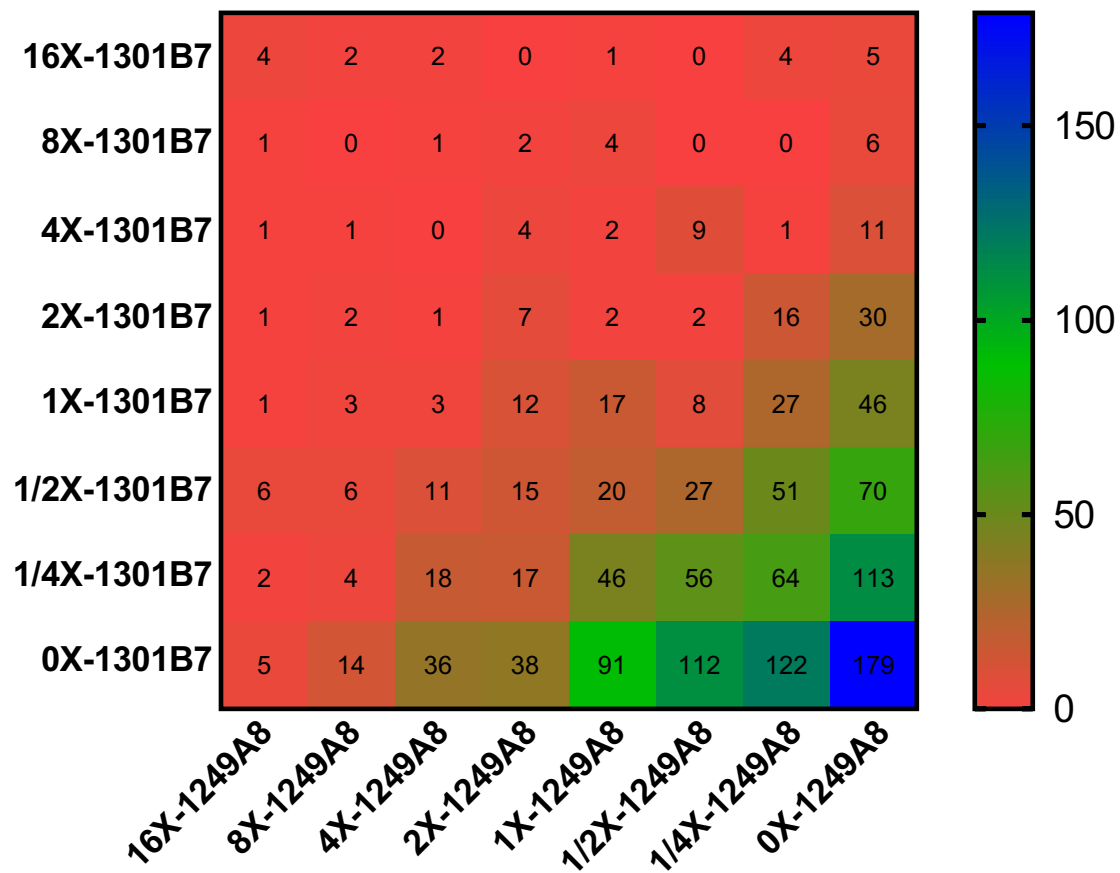

Figure S1

Supplement: Supplement 1 — Figure S1. Synergistic neutralizing activity of 1301B7 and 1249A8. A checkerboard plaque reduction neutralization test (PRNT) was used to evaluate potential synergistic neutralizing activity of 1301B7 and 1249A8 against Δ3a7b-Nluc. The vertical and horizontal axes represent the concentration of 1301B7 and 1249A8 (X-fold NT50), respectively. The heatmap represents the plaque count (spots) as a measure of viral infection, where a decrease in spots indicates neutralization. [file media-1.pdf]
